# Why clinicians overtest: development of a thematic framework

**DOI:** 10.1186/s12913-020-05844-9

**Published:** 2020-11-04

**Authors:** Justin H. Lam, Kristen Pickles, Fiona F. Stanaway, Katy J. L. Bell

**Affiliations:** Faculty of Medicine and Health, The University of Sydney School of Public Health, Edward Ford Building, A27 Fisher Rd, University of Sydney, Sydney, NSW 2066 Australia

**Keywords:** Medical overuse, Health service misuse, Overtest, Overtesting, Clinician

## Abstract

**Background:**

Medical tests provide important information to guide clinical management. Overtesting, however, may cause harm to patients and the healthcare system, including through misdiagnosis, false positives, false negatives and overdiagnosis. Clinicians are ultimately responsible for test requests, and are therefore ideally positioned to prevent overtesting and its unintended consequences. Through this narrative literature review and workshop discussion with experts at the Preventing Overdiagnosis Conference (Sydney, 2019), we aimed to identify and establish a thematic framework of factors that influence clinicians to request non-recommended and unnecessary tests.

**Methods:**

Articles exploring factors affecting clinician test ordering behaviour were identified through a systematic search of MedLine in April 2019, forward and backward citation searches and content experts. Two authors screened abstract titles and abstracts, and two authors screened full text for inclusion. Identified factors were categorised into a preliminary framework which was subsequently presented at the PODC for iterative development.

**Results:**

The MedLine search yielded 542 articles; 55 were included. Another 10 articles identified by forward-backward citation and content experts were included, resulting in 65 articles in total. Following small group discussion with workshop participants, a revised thematic framework of factors was developed:

- “Intrapersonal” – fear of malpractice and litigation; clinician knowledge and understanding; intolerance of uncertainty and risk aversion; cognitive biases and experiences; sense of medical obligation
- “Interpersonal” – pressure from patients and doctor-patient relationship; pressure from colleagues and medical culture;
- “Environment/context” – guidelines, protocols and policies; financial incentives and ownership of tests; time constraints, physical vulnerabilities and language barriers; availability and ease of access to tests; pre-emptive testing to facilitate subsequent care; contemporary medical practice and new technology

**Conclusion:**

This thematic framework may raise awareness of overtesting and prompt clinicians to change their test request behaviour. The development of a scale to assess clinician knowledge, attitudes and practices is planned to allow evaluation of clinician-targeted interventions to reduce overtesting.

**Supplementary Information:**

The online version contains supplementary material available at 10.1186/s12913-020-05844-9.

## Background

Overtesting has become a growing concern in contemporary healthcare, [1–3] with worldwide movements such as the Choosing Wisely, Less Is More, and Too Much Medicine campaigns bringing the issue to the fore [1, 4–7]. Despite this, awareness and understanding of the causes and consequences of overtesting amongst the public, patients and clinicians remains limited [3, 8, 9].

Medical tests are an important first step in the clinical management pathway, whether they are routine blood pressure measurements, blood tests, imaging studies or more specialised investigations. With the overall aim of healthcare being to prevent premature morbidity and mortality [10], it follows then that the value and rationale for performing tests lies in their ability to improve patient health outcomes [11] through detection and/or monitoring of disease and subsequent treatment. These health outcomes may be clinical, emotional, social, cognitive or behavioural [10].

Tests that are done without improving health outcomes (as defined above), may be examples of overtesting. Overtesting includes unnecessary medical tests in both asymptomatic and symptomatic people, [2] where testing does not improve clinical decision making (clinical utility), or health outcomes (clinical effectiveness) [12]. Overtesting of screening and diagnostic tests may occur where there is an unfavourable balance of benefits to harms [13] and where there would be little or there would be no consequences to the patient from not performing the test [12]. Overtesting of monitoring tests may occur where the tests have poor measurement properties, and/or are done overly frequently (low signal: noise ratio) [14].

Potential harms from overtesting arise through misdiagnosis, false positive results, false negative results and overdiagnosis, where people are labelled as having a “disease” for a condition that would not have caused them harm if it were left undetected and untreated [1, 5, 6, 8, 9, 15–18]. More often than not, this initiates a further cascade of unnecessary investigations and treatment [1, 15, 16, 19–22]. Individuals may be affected physically, psychologically and financially [8, 9, 15, 16, 23] and the use of finite healthcare resources prevents their redeployment to others who would have benefited from tests, treatment and other interventions [8, 15, 16, 23]. These unintended consequences increase proportionately with the degree of overtesting [12].

Clinicians are the “gatekeepers” [24] to accessing further healthcare, and are ultimately responsible for requesting medical tests, making diagnoses and offering treatment and/or further tests. Their stewardship of medical testing is key to limiting overtesting and preventing the cascade of harms that may result.

To date, there has been no systematic synthesis of published evidence exploring factors that influence clinicians to overtest. In this narrative literature review and workshop discussion with experts at the Preventing Overdiagnosis Conference 2019, we aimed to identify and create a thematic framework of important factors that may influence clinicians to overtest. The results of this study will inform further planned research to develop a scale assessing clinician knowledge, attitudes and practices around overtesting. The new scale will be used to guide the development of clinician-targeted interventions to prevent overtesting [25], and to measure their impact on testing behaviour.

## Methods

We conducted a narrative review of peer-reviewed literature using Boell’s [26] hermeneutic approach characterised by a systematic but flexible and iterative search strategy. A narrative review aims to summarise, interpret and critique available literature based on the authors’ own understanding, or pre-existing theories and models available [27–29]. Through an initial non-systematic search of the literature, we identified recurrent themes and developed inclusion and exclusion criteria and the search strategy.

We searched MedLine from inception to April 2019 for published articles using MeSH terms and keywords related to the concepts of “overtesting”, “physician” and “attitude to health” [**Supplementary File** 1]. We included articles that focussed on overtesting or overscreening and explored factors influencing clinicians’ test requesting behaviour. We did not limit our search to specific conditions, tests or specialties. To supplement the MedLine search, we identified further articles through forward and backward citation and consultation with content experts in overdiagnosis. We excluded articles that focussed on overtreatment, unnecessary treatment or factors influencing clinicians’ treatment decisions.

Two authors (JL and KB) independently screened titles and abstracts. Where either author considered the paper potentially relevant, the full text was retrieved. Two authors screened the full text for inclusion in the review (JL screened all full text, and KB, FS and KP each screened one third). One author (JL) undertook inductive thematic analysis [30] of the included studies to identify distinct factors influencing clinicians’ decisions to order tests. These were initially categorised in a thematic framework of “direct/intrinsic” factors and “indirect/extrinsic” factors. A second author reviewed included studies and added additional factors to the framework (KB, FS and KP each reviewed one third of included studies) **[Supplementary File** **2****].**

The resulting framework of “direct/intrinsic” and “indirect/extrinsic” factors was presented at a workshop at the Preventing Overdiagnosis Conference in Sydney, December 2019. The workshop used small group discussion to receive feedback on the initial framework, and to elicit further factors that may influence clinician test ordering behaviour not identified through the narrative review. Basic demographic information was collected from workshop participants with their consent. There were five-six participants in each small group, with discussion facilitated by one of the authors. Discussions were audio-recorded and transcribed for further analysis by the authors to refine the thematic framework developed from the narrative review.

## Results

### Results of narrative review

**Fig. 1:**
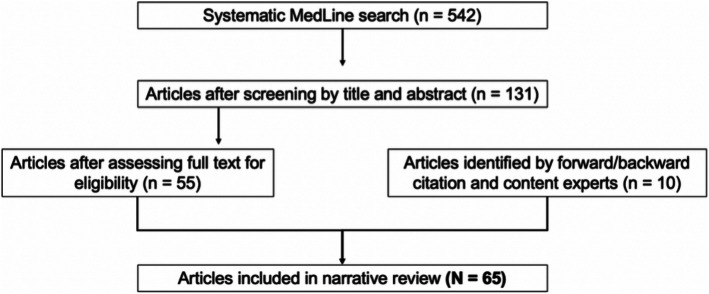
Study flow diagram

A total of 542 articles were retrieved following the search of MedLine in April 2019. 55 articles were included after screening by title and abstract, and application of inclusion/exclusion criteria to full text articles. A further 10 articles identified through forward and backward citation and by content experts were included, resulting in a total of 65 articles in the review. **[**Fig. 1**].**

### Direct/intrinsic factors

#### Fear of malpractice and litigation

Thirty-four articles cited fear of malpractice and litigation as a cause for unnecessary testing and diagnosis. A number of studies demonstrated a positive correlation between likelihood of ordering imaging tests and level of litigation concern [31–34]. Hoffman and Kanzaria [35] referred to a survey of United States (US) emergency physicians where 97% of respondents ordered advanced imaging tests due to fear of litigation and missing a low probability diagnosis despite feeling the tests were unnecessary. Sanabria et al. [36] showed that pathologists concerned about litigation tended to lower implicit disease thresholds for indeterminate and malignant tumour diagnoses. Conversely, a number of studies deemed malpractice not to be a major driving factor for ordering tests [37–39].

#### Clinician knowledge and understanding

Twenty-five articles highlighted unnecessary testing as a compensatory measure for lack of knowledge and understanding. For most studies, this related to lack of knowledge and understanding of the drivers of overuse, the natural history of disease, and appropriate management pathways. Cardiologists and non-cardiologists [37] reported that greater training and experience helped them understand when to perform an echocardiogram and in which patients as they “understand the natural course of disease” [37]. Through their novel reflective writing program, Caverly et al. [40] demonstrated that greater cognisance of the drivers of overuse can positively impact clinical decision making and test ordering behaviour through empowerment to identify, discuss and avoid overuse.

Wegwarth et al’s [41] randomised trial assessing 412 primary care physicians’ understanding of common screening statistics found that lack of knowledge and understanding of significance of test properties and results also encourages overtesting. Physicians were more likely to recommend a test when presented with evidence based on increased 5 yearly survival rates (which tend to overestimate the benefit of early detection and treatment because of lead time bias and overdiagnosis) as compared with reduced mortality rates (which give an unbiased estimate of benefits) [41].

#### Intolerance of uncertainty and risk aversion

This factor was cited in 24 articles. In the 2017 American Board of Internal Medicine (ABIM) Foundation survey, [42] among the commonest reasons for ordering low value tests were desire to reduce uncertainty (84%) and “just to be safe” (78%) [39]. Egerton-Warburton et al. [43] found that over half of 1029 emergency physicians in the study reduced their implicit “test threshold” well below the explicit threshold set using empirical data on test accuracy and risk of harm from the disease, due to their discomfort with diagnostic uncertainty [43]. Coon et al. [23] also highlighted the “shotgun approach” [23] of ordering a broad range of tests and hoping for a positive result somewhere, which is often used in situations of diagnostic uncertainty. A US survey [44] of primary care providers’ and gynaecologists’ breast cancer screening practices showed that those with greater levels of anticipated regret in missing serious disease were more likely to recommend mammography [44]. However, in assessing factors influencing tendency to order imaging using hypothetical scenarios, Kini et al’s [33] survey of cardiologists and general practitioners did not find a significant association between risk aversion and tendency to order cardiac stress tests and echocardiography (likely due to their small sample size) [33].

#### Cognitive biases and previous experiences

Unnecessary testing often occurs as a result of cognitive biases; two significant cognitive biases were found in 12 articles**.** The first was “availability bias” [4, 45] which occurs when the likelihood of future events is estimated based on ease of recall of similar events. Closely associated is the impact of previous experience of clinical events. Clinicians with recent negative experiences or “recent medical blunders” [46] were more likely to adopt an aggressive approach to diagnostic testing [10, 38, 45, 46]. Gyftopoulos et al. [47] and Sanabria et al. [36] also suggested that positive experiences from test ordering can increase the likelihood of ordering further tests in the future.

The second cognitive error is “representative bias” [10, 15, 48] or “base rate neglect” [10, 15, 43] which occurs when failing to take into account pre-test probability in estimating post-test probability in the setting of a positive test result [15, 21, 43]. By overinterpreting positive results, clinicians are more likely to order further tests. In Austin’s [48] survey, it was found that 10% of physicians had incorrectly deemed positive predictive value to be the same between screening (where pre-test probability is low) and diagnostic (where pre-test probability is high) tests.

#### Sense of medical obligation

This factor was cited in 6 articles. Testing based on a sense of medical obligation was driven by the clinician’s need to show that at least something was being done for the patient [46, 49]. Simmonds et al. showed that when faced with the decision as to whether to disclose a diagnosis of clinically inconsequential chronic kidney disease, some general practitioners felt morally bound to respect patient autonomy and disclose results so as to allow the patients to make their own health decisions [50].

### Indirect/extrinsic factors

#### Pressure from patients and doctor-patient relationship

This was reported in 29 articles. Van der Weijden et al. [46] noted that not only were anxious patients more likely to request tests, but general practitioners conscious of long term relationships with patients were more likely to meet these requests. In the 2014 and 2017 American Board of Internal Medicine survey, [42] a large proportion of participants cited desire to keep patients happy, patient’s insistence, and the idea that patients should make the final decision, as reasons for ordering tests. Gogineni et al. [51] and Griffith et al. [52] showed that clinicians were more likely to acquiesce to patient demands for tests the clinician judged as unnecessary if patients threatened to see another clinician. Conversely, He [38] and Siedlikowski et al. [53] found that the better the relationship, the less likely patients were to demand unnecessary tests, and the less likely doctors would be to order unnecessary tests.

#### Guidelines, protocols and policies

Interpretations and attitudes toward guidelines, protocols and policies were found to significantly influence test ordering behaviour in 21 articles. Akerman et al. [54] found that that there was a drop in prostate screening rates from 91.7 to 80.4% when new recommendations from the Canadian and United States Preventive Services Task Force were released following evidence of little net benefit from screening. A number of studies showed that non-existent or discordant guidelines can result in overtesting [45, 53, 55–57]. However, some studies demonstrated that the use of protocols resulted in inappropriate use of tests and overtesting [36, 37, 58]. Alber et al. [49] and Bishop et al. [59] found that doctors based their test ordering decisions on individual patient cases, using guidelines “as a guide rather than strict rules” [59]. This resulted in variation in test ordering behaviour, with some more likely to test while others less likely [49, 59].

#### Financial incentives and ownership of tests

This was reported in 21 articles. While financial incentives refer to the remuneration received from directly ordering a test, ownership of tests results in a personal, vested interest from more tests being performed, as well as remuneration on a larger scale. Moynihan and Doust [1] and Sanabria et al. [36] refer to the concept of “physician induced demand” [21] whereby physicians can order return visits and perform diagnostic tests when indications are vague or controversial. Pickles et al. [60] showed that Australian general practitioners (fee-for-service health system) were more likely to order prostate specific antigen tests than their United Kingdom counterparts (no fee-for-service health system). Fonesca et al. [37] suggested that clinicians working in private healthcare settings were more likely to be driven by economic incentives in regards to test ordering. Physicians who own imaging equipment engage in more testing with similar clinical outcomes, [15] implying a financial conflict of interest as a driver of overtesting. Conversely, general practitioners in Simmonds et al’s [50] study provide a case study of how clinicians may resist financial incentives to test when they are motivated and committed to preventing overtesting. GPs in this study resisted financial incentives to keep a register and monitor patients with stage 3 chronic kidney disease, as this conflicted with their beliefs around the meaning of mild reduction in kidney function and were concerned that “they’re creating an illness that doesn’t exist [50].

#### Pressure from colleagues

Pressure from other clinicians was as a driver of unnecessary testing in 13 articles. Fonesca et al’s [37] cardiologists admitted to ordering echocardiograms when colleagues deemed it necessary despite themselves thinking otherwise. D’Souza et al. [61] also showed that junior doctor test ordering was significantly influenced by their peers, colleagues and supervisors. Siedokowski et al. [53] found that as many as 89.6% of physicians would order a screening test they would not have otherwise ordered if specialists had recommended the test.

#### Time constraints

Time constraints for clinical assessment was another factor behind excessive testing in 13 articles. A number of studies recognised the time pressures in the work environment which limits time with patients and encourages physicians to provide a test just to expedite the clinical encounter [34, 46, 59, 62, 63]. Ellen and Horowitz’s [64] survey of Israeli nurses showed that more than half felt that giving physicians more time to discuss alternative tests would reduce overuse. On the other hand, Murphy et al. [65] highlighted that some general practitioners felt doing an annual general check-up on otherwise healthy patients represented a waste of their limited time that was better spent attending to sick patients.

#### Availability and ease of access to tests

This factor was reported in 10 articles. Tests were more likely to be ordered when logistically easier, for example, during day shifts as opposed to evening or night shift, [64] when in closer proximity, [21] when there was little resistance in test ordering [47] or when able to order from desktop devices [46]. Fonesca et al. [37] also noted that waiting times and patient physical mobility affected likelihood of echocardiogram test ordering. Having the available technology and equipment was also a key predictor of testing ordering, such was the case in tertiary hospitals [38].

#### Pre-emptive testing to facilitate subsequent care

Reported in 10 articles, uncertainty about what future tests would be required, a desire to avoid delays in a patient’s care pathway and testing to establish a “baseline” were recognised as causes for potentially unnecessary testing. Amongst Irish interns, Flynn et al. [55] noted that almost half requested tests they felt their consultant would want, to ensure that cases were never cancelled due to a lack of data. Sears et al. [34] found that 76% of physicians felt they couldn’t refer a patient to a specialist without having magnetic resonance imaging done first [34]. Similarly, Alber et al. [49] highlighted that some general practitioners considered “medical overuse in inpatient care as a welcome diagnostic work-up and baseline for the subsequent outpatient care” [49] with Munce et al. [66] noting that some family physicians ordered bone mineral density tests in asymptomatic women at menopause to obtain a “baseline” [66].

#### Contemporary medical practice and new technology

Four articles highlighted that in contemporary medical practice, emphasis and even reliance on technical tests with a relatively lower priority given to history taking and physical examination, contributes to overtesting [7, 36, 37, 49]. Lysdahl and Hoffman [67] also showed that improved radiological technology was a major cause of overall increased investigation volume that, in turn, predisposed to unnecessary investigation.

### Results of expert group discussion

There were 15 participants at the Preventing Overdiagnosis conference workshop. All but one participant were either academics/researchers or medical doctors and most had been working for at least five years. **[Supplementary File** **3****]**.

Participants generally agreed with the importance of factors identified through the narrative review. A number of additional suggestions were made and subsequently integrated into the existing framework. Physical vulnerabilities (such as being fatigued or hungry) and language barriers were included in “time constraints” as participants felt that such motivational factors for test ordering were based on the desire to reduce patient-contact time. The notion of wanting to fit into the existing medical culture was included in “pressure from colleagues”.

Participants believed that the initial framework of “direct/intrinsic” and “indirect/extrinsic” factors was too dichotomous and artificial, and did not adequately illustrate the overlap and interaction between all the factors. Participants considered that most of what was conceptualised as “indirect/extrinsic” is actually moderated by the “direct/intrinsic” values and perceptions of the individual clinician, and that neither of these properties are fixed but change over time. Participants suggested modifying the proposed framework to better highlight the complex relationship between factors influencing clinician test ordering behaviour to aid in the development of the scale measuring clinician knowledge, attitudes and practices around overtesting.

Based on this feedback, we revised the initial framework to incorporate the factors in a new framework of: “intrapersonal”, “interpersonal” and “environment/context” factors**.** We believe that these categories provide a more useful framework for informing construction of a scale to measure clinician knowledge, attitudes and practices on overtesting. The total number of articles reporting on each factor and quotes (from participants involved in qualitative interview studies) reflecting clinician attitudes and reactions are provided in (Tables 1, 2 and 3). Detailed analysis of workshop data is available via **[Supplementary File** **4****]**.

**Table 1:** “Intrapersonal factors” affecting clinician test ordering behaviour, with number of articles and quotes from articles

Factor	Articles	Illustrative quotes
Fear of malpractice and litigation	34 articles [1, 2, 7, 21, 23, 31–39, 42, 45–47, 49, 59, 60, 62–64, 68–77]	“You are so open for being sued by anything but it’s very easy to want to lean towards the screening everyone … I definitely think it’s hard not to think legally” [45] “Once the issue has been raised, it is difficult to back away unless you are 100% because you are responsible if you are wrong” [45] “I’m often a bit defensive...I guess that’s partly that legal thing” [45] “I think the whole medical-legal thing also makes people more inclined to CT [computed tomography] someone even if they have a pretty low suspicion just ‘cause no one wants to be sued” [47] “I think litigation is a problem; you miss one neck... fracture or bleed in the brain you are going to court” [62]
Clinician knowledge and understanding	25 articles [2, 3, 5, 21, 37, 40, 41, 43, 45–47, 49, 50, 58, 62, 64–66, 72, 73, 78–82]	“How much work [laboratory testing] is, how much it costs, how much normal results can fluctuate, things like that, I think we know very little about that” [3] “Nothing can really go wrong [with overutilization]” [3] “You understand the natural course of disease and the point in time at which you have to make a decision to do something different” [37] “When I’m admitting a patient or doing clinical work, it’s kind of affected my thought process to where I think a little bit more about ‘do I really need to get this test?’, ‘will it really change management?’, ‘could it potentially be harmful to the patient?’” [40] “Those like statistical issues don’t apply to the individual...because...they make their decisions on a set of complex, but perhaps irrational basis, you know, anxiety and...” [45] “Yeah, so, I hate the D-dimer. I understand its utility. I think that too many D-dimers are sent... I think the decision to get a CTPA [computed tomography pulmonary angiogram] should be based on a clinician’s clinical reasoning plus or minus the criteria, plus or minus a D-dimer” [47] “As I said, a patient without previous medical history, without symptoms. In this case, I have never auscultated a lung and thought: “Thank god I listened to that lung.” I mean, what do you expect from a healthy patient when you auscultate the lung? A healthy lung” [49] “GPs may be playing a good game and saying I’m not going to bother this patient with having a GFR [glomerular filtration rate] of 59 because I know that although it qualifies as CKD [chronic kidney disease] 3 it’s not gonna make any difference to how I manage that patient and I think that’s good medicine” [50] “When you have no idea what’s going on, so it gives you something to hide behind” [62] “‘Should be tailored according to family history, previous issues, lifestyle and previous findings. Need to explain the limitation of check-ups” [65]
Intolerance of uncertainty and risk aversion	24 articles [1–3, 7, 21, 23, 32, 33, 35, 37, 39, 42–44, 46, 47, 49, 53, 64, 69–71, 73, 83]	“Lab testing is often only done for the doctor’s peace of mind.” [3] “I am worried if they don’t have a full assessment and I miss something that it is going on with their heart that is not apparent because ECGs [electrocardiograms] and clinical examinations are not very precise” [37] “What if it couldn’t wait? How would you know it won’t affect them?” [45] “You’re sitting there with someone who has a sudden-onset splitting headache, but otherwise you see nothing alarming … A CT scan for an acute headache. Even if the pre-test chance is 0.01. He does it anyhow. They have much more certainty than we do.” [46] “You have to be self-confident in not doing something” [49]
Cognitive biases and experiences	12 articles [15, 21, 36–38, 45–48, 60, 84, 85]	“‘There might be a bias to a situation where some doctors missed an important finding, when they were a junior doctor, so they always do scans because they are worried that something might happen like years ago” [37] “It’s certainly a—hard to be, treating dying people who are young and not to worry about all of this and I, but I try not to change my practice based on my own personal experience of one or two people dying of prostate cancer” [45] “If you’ve ever experienced something like that, you can be sure that you’ll send patients with vague complaints for further testing much faster. Absolutely” [46] “I would say that my clinical experience highly in- fluences my ordering … sometimes I feel a certain way about a patient even though they don’t fit a certain profile and I’ll end up doing something additional for them” [47] “The initial thing was PSA [prostate specific antigen] is useful and that has basically stuck in my head, that PSA testing is useful” [60]
Sense of medical obligation	6 articles [16, 45–47, 49, 50]	“To not screen somebody, I don’t know, it seems cruel, it’s cruel and irresponsible... to not at least make an attempt to avoid the misery of a person getting prostate cancer, to me, seems unbelievably cruel” [45] “We have to diagnose them if they have a problem” [45] “Some GPs mentioned their frustration at not being able to offer the patient something useful, at the feeling of empty hands, owing to the lack of a diagnostic or therapeutic plan for patients presenting with unexplained complaints. A test request symbolises a serious attempt to deal with the patient’s complaint” [46] “If it’s on your radar … you’re almost honor-bound to do the study of choice” [47] “‘Action’ dogma of doing anything possible for the individual patient” [49] “My personal policy I would always disclose...generally speaking I would always explain the diagnosis” [50]

**Table 2:** “Interpersonal factors” affecting clinician test ordering behaviour, with number of articles and quotes from articles

Factor	Articles	Illustrative quotes
Pressure from patients and doctor-patient relationship	29 articles [2, 21, 34, 37–39, 42, 45, 46, 49, 51–53, 59–67, 70, 71, 73–75, 86, 87]	“It can reduce the anxiety and prevent representations to the hospital, helping to keep them from coming in with chest pains” [37] “Now she had problems with her feet and arms, morning stiffness, pain in the joints. But there was no redness, no swelling, wasn’t warm, functioning was good. But she was still uneasy. I had to confirm this to her with a blood test, otherwise the discussion would go on and on” [46] “But the GP lives in the community, has to continue caring for the patient. If you really mess things up, so that the patient switches to another doctor, that’s what affects me” [46] “Patients come in and they say, ‘Oh, I have this, and I want a CT scan done.’ They’ll tell you what they want done” [59] “If we order more tests and we make sure we have every test ordered that might possibly be needed, the patient’s happy and leaves in their ED [emergency department] stay” [59] “So they see it as their right to have it” [60] “There is a demand from patients for testing or medication or imaging that they’ve read about or they feel that they should get in order to be satisfied that they’ve been adequately cared for” [62] “Patients absolutely drive test ordering...” [62] “I guess I do it because...I want my patients to perceive that I practice good medicine...you do have to be seen to be proactive” [63] “Can improve relationship between patients and doctor” [65] “Check-ups are largely patient driven secondary to media/public health generated anxiety” [65] “I’ll say “well you just had one two years ago, you’re on treatment, it was stable from the year before, and I don’t think you need one” … what does usually happen is that they usually win” [66]
Pressure from colleagues (and medical culture)^a^	13 articles [3, 36, 37, 45, 46, 49, 53, 55, 59–61, 69, 86]	“Well, often the supervisor just says to run some tests, and I just accept that without question” [3] “I recently ordered a lipase, but then the gastroenterologist called me and said: in this hospital, we always combine it with an amylase” [3] “If an experienced cardiology colleague says we should do another echo, I would not feel strong enough to say no” [37] “If the neurologist had written, “There’s nothing the matter” ... But how must I say “you have to accept it” if the neurologist says that perhaps the patient should be looked at by someone else” [46] “If I get a letter from the diagnostic centre with the comment “You request 10% more than the average GP in Maastricht”, then you get critical. You wonder if we should wait a bit longer with this patient” [46] “If you’re not going to order it, the next doctor will” [59] “He would see the cardiologist every three months and would get a stress test every year...When he came to see me...I had to tell him ‘I don’t think that that’s necessary” [59] “A lot of tests get done that probably don’t need to get done because our residents are afraid of not ordering something because they’ll disappoint us” [59]

Following expert focus group discussion:

^a^ “medical culture” was grouped with “pressure from colleagues”

**Table 3:** “Environment/context factors” affecting clinician test ordering behaviour, with number of articles and quotes from articles

Factor	Articles	Illustrative quotes
Guidelines, protocols and policies	21 articles [33, 36, 37, 45, 47, 49, 50, 53–60, 62, 64, 66, 71, 72, 86]	“There are situations where I’ve ordered an echo when I otherwise would not have because guidelines mandated” [37] “I think there’s more, as much as we’ve developed these decision rules—I think there’s a lot to be said about just experience” [47] “I think people are wary of practicing not in line with that and then they have potential then for criticism” [60] “There’s plenty of guidelines, but they’re all different and there’s nothing official...there’s no hard and fast rule” [60] “Because I work in a teaching practice, my residents are very devoted to guidelines. A lot of them are driven by the more recent guidelines” [66]
Financial incentives and ownership of tests	21 articles [1, 2, 7, 15, 21, 23, 36–38, 42, 49, 50, 55, 60, 64, 65, 69, 70, 73, 83, 85]	“Identifying more disease means more business” [21] “If I went around having my 10 min discussion with all my patients about why not to do PSA testing, I will make less money than [a GP] who does the 30 s— here Jack, that’s a good idea, here, have the PSA test” [60] “To be perfectly honest, I only do it because of patient expectation as a business decision, not as valid evidence based medicine” [65] “A lucrative source for the private hospitals” [65]
Time constraints, (physical vulnerabilities and language barriers)^a^	13 articles [3, 23, 34, 37, 46, 47, 53, 59, 62–65, 70]	“Some days patients want tests that I feel are not necessary but I want to avoid discussions or I’m tired and I will order tests anyway” [37] “If you had enough time to do a thorough history-taking of all these people … People would say ‘“I think I’ve been well understood, listened to, and examined”, and need far fewer further investigations. But that is much too time consuming” [46] “You see many exams ordered, “Rule out PE [pulmonary embolus],” and that’s all that you have … we often just go ahead and do the exam, to be honest, because it ends up creating a lot of lost time” [47] “They do a lot of catscans because they don’t have time to observe patients … work them up, get them out the door” [59] “If I’m really busy and I have ten people in the waiting room, and if I feel pressured and overwhelmed, I can say,‘Yep, here is a requisition for the MRI [magnetic resonance imaging], let’s get it done and move along” [62] “A major concern that it could increase workload which would diminish time for treating ill patients” [65]
Availability and ease of access to tests	10 articles [3, 21, 36–38, 46, 47, 57, 64, 67]	“Checking boxes on the lab form, I often go, let’s do this one too, and that one” [3] “When you’re ordering lab tests, it is easy to just order some more tests” [3] “The patient is already being sent for another test to the diagnostic centre, which creates a low threshold for doing more testing … so why not?” [46] “I think for any test if it’s very, very available and it’s fast and it’s easy to do and it doesn’t take a lot of time and there’s more turnaround on the report— then we’re just more likely to use it more.” [47] “It would probably be valuable to make the process less convenient because the threshold is so low to order CTs” [47]
Pre-emptive testing to facilitate subsequent care	10 articles [34, 37, 49, 55, 59, 60, 62, 65, 66, 82]	“I am glad that I can refer to something … And you could describe that as medical overuse to some extent. Because we are talking about tests which were not totally urgent or rather luxurious given the specific symptoms at that time. But it can be really helpful to have this reference point” [49] ““We order tests because we feel we have to get everything up front, because it’s just too painful to do things too slow, to do things as a series” [59] “People are used to sort of being screened...so we’re tacking this onto the discussion basically” [60] “They will tend to steer on the side of getting a test, even though it may be unnecessary, because they fear they will not be able to get the patient referred” [62] “You only realize the importance once you do it—the yield of significant results is surprising” [65] “Often I’m doing [BMD tests] at menopause time in a woman’s life when things sort of come up. I get a baseline maybe at menopause” [66]
Contemporary medical practice and new technology	5 articles [7, 36, 37, 49, 63]	“There is less emphasis on clinical examination. Nowadays we hear murmurs, and we try to quantify their severity which leads straight to ordering an echo … However, this can result in overuse of imaging” [37] “The greatest challenge will be to put more emphasis on history taking and physical examination again … This is the prerequisite to avoid further unnecessary investigations” [49]

Following expert focus group discussion:

^a^ “physical vulnerabilities” and “language barriers” were grouped with “time constraints”

## Discussion

This narrative literature review, and subsequent small group discussion with experts in preventing overtesting, highlights the myriad of factors that influence a clinician’s decision to order medical tests, often unnecessarily or against evidence-based recommendations. We present a framework for understanding factors that influence clinicians to overtest. These were grouped within a final framework of “intrapersonal”, “interpersonal” and “environment/context” factors. The most commonly cited factors were “fear of malpractice and litigation”, “pressure from patients and doctor-patient relationship”, “clinician knowledge and understanding”, “guidelines, protocols and policies” and “intolerance of uncertainty and risk aversion”.

We are aware of no other systematic evidence synthesis exploring in detail the factors that influence clinician decisions to order unnecessary tests. Pathirana’s [7] analysis article explored the drivers of overdiagnosis which are closely linked to that of overtesting and mapped them to potential solutions but did not focus on the drivers from a clinician perspective. Siedlikowski et al. [53] and Sharma et al. [57] identified a number of factors influencing clinicians’ recommendations for mammography screening, but not test requesting behaviour more broadly.

One intended outcome of this thematic framework is that it will help clinicians become more aware of their test requesting behaviour, and that this self-reflection may be a critical first step to the behavioural changes needed to prevent overtesting. The grouping of “intrapersonal”, “interpersonal” and “environment/context” clearly reflect the broad range of pressures faced by clinicians on a daily basis. For example, with societies becoming increasingly punitive [68] for errors of omission rather than commission, [23] clinicians are understandably motivated to order tests as a strategy for lowering legal risk and avoiding the financial and emotional consequences of litigation [35]. Moreover, pressures and demands from patients is a growing issue in modern healthcare [51] partly driven by information publicly available on the Internet [37, 52] and social media [21, 60]. Such information may be low quality, unreliable or incomplete, [52] and patients may have unrealistic or ill-informed expectations [88]. The culture of “shame and blame” [35] and medical education that instils fear of uncertainty [24] further encourages overtesting. Through greater awareness of their test requesting behaviour, clinicians may be more likely to only request tests when these may helpfully inform their clinical decision making, and ultimately improve health outcomes [12].

This framework may also provide a basis for developing interventions to prevent overtesting by targeting a specific group of factors and preferably all groups in the framework rather than individual factors alone. Too narrow a focus, for example, increasing clinician knowledge without taking into account the impact of financial incentives, may cause an intervention to be less effective.

The results of this study and framework will also be used to inform the development of a scale to assess clinician knowledge, attitudes and practices around overtesting and to measure the impact of clinician-targeted interventions to prevent overtesting and its unintended consequences.

Strengths of our study are the systematic approach to retrieving the literature, use of at least two authors at each step of the review process and rigour in thematic analysis of the data with multiple iterations through discussion with all authors. We were able to engage with content experts at the Preventing Overdiagnosis Conference workshop, and revise the initial proposed framework. Our final framework is not only more useful for measuring clinician-targeted strategies and interventions, but also explores the complex interplay of factors that occurs in real clinical practice. A limitation of our study was that we searched only one database which may have limited the number of articles included. However, given the richness of data and complexity of the thematic framework, it seems unlikely that we missed important factors that influence test ordering behaviour. This is also supported by the fact that the small group discussion did not generate any new, distinct factor groups.

## Conclusions

Although medical tests are integral to clinical management, clinicians have the ability and responsibility to limit overtesting and to prevent harm to patients and the healthcare system. This thematic framework describes factors that influence decisions to request tests as a first step in developing clinician-targeted interventions to reduce overtesting.

## Supplementary Information

**Additional file 1 Supplementary File 1** Search Strategy: includes all the search terms used and number of articles retrieved for the narrative literature review.

**Additional file 2 Supplementary File 2** Initial Thematic Framework: Excel Spreadsheet of articles included in the narrative literature review, thematic analysis and incorporation into initial framework of direct/intrinsic and indirect/extrinsic factors.

**Additional file 3 Supplementary File 3** Demographic Characteristics: table including demographic characteristics of PODC workshop participants.

**Additional file 4 Supplementary File 4** PODC Workshop Data: table detailing audio-recorded comments (de-identified) from workshop participants regarding each of the factors affecting clinician decisions to overtest.

## Data Availability

The datasets used and/or analysed during the current study available from the corresponding author on reasonable request.

## Abbreviations

ABIM: American Board of Internal Medicine
CKD: Chronic kidney disease
CT: Computed tomography
CTPA: Computed tomography pulmonary angiogram
ECG: Electrocardiogram
ED: Emergency department
GFR: Glomerular filtration rate
GP: General practitioner
MRI: Magnetic resonance imaging
PE: Pulmonary embolus
PODC: Preventing Overdiagnosis Conference
PSA: Prostate specific antigen
US: United States
