# Supplementary material for: Why clinicians overtest: development of a thematic framework
Source: BMC Health Serv Res. 2020 Nov 4;20:1011. doi: 10.1186/s12913-020-05844-9 (PMC7643462; doi:10.1186/s12913-020-05844-9)
Supplement: Supplementary file 1 — Additional file 1 Supplementary File 1 Search Strategy: includes all the search terms used and number of articles retrieved for the narrative literature review. [file 12913_2020_5844_MOESM1_ESM.pdf]

[Supplementary File 1] Search strategy

# ▲	Searches	Results	Type	Actions
1	exp Medical Overuse/	6305	Advanced	Display Results | More
2	overdiagnos$.mp.	3755	Advanced	Display Results | More
3	over diagnos$.mp.	1287	Advanced	Display Results | More
4	overtest$.mp.	74	Advanced	Display Results | More
5	over test$.mp.	353	Advanced	Display Results | More
6	1 or 2 or 3 or 4 or 5	11309	Advanced	Display Results | More
7	exp Physicians/	128157	Advanced	Display Results | More
8	physician$.mp.	533965	Advanced	Display Results | More
9	clinician$.mp.	207853	Advanced	Display Results | More
10	doctor$.mp.	119116	Advanced	Display Results | More
11	7 or 8 or 9 or 10	808467	Advanced	Display Results | More
12	exp Health Knowledge, Attitudes, Practice/	102425	Advanced	Display Results | More
13	exp "Attitude of Health Personnel"/	150317	Advanced	Display Results | More
14	attitude$.mp.	397625	Advanced	Display Results | More
15	perce$.mp.	1225432	Advanced	Display Results | More
16	view$.mp.	440617	Advanced	Display Results | More
17	understand$.mp.	1033085	Advanced	Display Results | More
18	12 or 13 or 14 or 15 or 16 or 17	2812625	Advanced	Display Results | More
19	6 and 11 and 18	542	Advanced	Display Results | More
