# Supplementary material for: Why clinicians overtest: development of a thematic framework
Source: BMC Health Serv Res. 2020 Nov 4;20:1011. doi: 10.1186/s12913-020-05844-9 (PMC7643462; doi:10.1186/s12913-020-05844-9)
Supplement: Supplementary file 3 — Additional file 3 Supplementary File 3 Demographic Characteristics: table including demographic characteristics of PODC workshop participants. [file 12913_2020_5844_MOESM3_ESM.pdf]

[Supplementary File 3] Demographic characteristics of 15 workshop participants at the Preventing Overdiagnosis Conference, Sydney, 2019.

Gender	Age (years)	Profession	Duration in workforce (years)	Country	Setting	Healthcare Sector
F	30-39	Medical doctor<br>Academic/researcher	5-10	Europe	Metropolitan	Public
F	50-59	Medical student	<5	Australia	Metropolitan	-
M	40-49	Academic/researcher<br>Other	>20	Australia	Metropolitan	Private
F	60-69	Academic/researcher	10-15	Australia	Rural	-
F	30-39	Medical doctor<br>Other	5-10	Australia	Both	Both
M	30-39	Academic/researcher	5-10	Australia	Metropolitan	-
F	60-69	Medical doctor	>20	Europe	Metropolitan	Public
F	50-59	Other	15-20	Australia	Both	Public
F	60-69	Academic/researcher	5-10	Australia	Metropolitan	-
M	60-69	Medical doctor	>20	Denmark	Both	Both
M	30-39	Medical doctor	≤5	Denmark	Metropolitan	Public
M	50-59	Medical doctor (nephrology)	≥20	Colombia	Both	Both
F	40-49	Academic/researcher	15-20	Canada	Metropolitan	Public
F	50-59	Medical doctor (GP/family physician)	≥20	Australia	Metropolitan	Private
F	60-69	Medical doctor (GP/family physician)	≥20	Australia	Rural	Both

F = female ; **M** = male
