# Supplementary material for: Why clinicians overtest: development of a thematic framework
Source: BMC Health Serv Res. 2020 Nov 4;20:1011. doi: 10.1186/s12913-020-05844-9 (PMC7643462; doi:10.1186/s12913-020-05844-9)
Supplement: Supplementary file 4 — Additional file 4 Supplementary File 4 PODC Workshop Data: table detailing audio-recorded comments (de-identified) from workshop participants regarding each of the factors affecting clinician decisions to overtest. [file 12913_2020_5844_MOESM4_ESM.pdf]

**INTRAPERSONAL FACTORS**	Intolerance of uncertainty and risk aversion	• Holding others’ intolerance of uncertainty<br>o *So they can’t tolerate maybe that this patient doesn’t have something serious. But they’ll request the test so that I can report it and say it’s fine. So I then have to kind of hold that, which I’m happy to do but it, it does make you feel that a lot of your work is futile…But someone has to hold that uncertainty* (W1F)<br>o *I can have certain tolerance of uncertainty but if the patient does not have a tolerance of uncertainty, therein lies the problem* (W2J)<br>o *(At the hospital) you might be unsure of something but if the senior doctor, one of the other departments have a different tolerance… it’s going to change your (?) to* (W2J)<br>o *So someone might come in with a very vague symptom, which could be something serious. So that then drives them into radiology. We then say it’s fine and they can discharge the patient, just get them out* (W1F)<br>• A general rule in medicine is/was *If uncertain, do it. There’s not a choice. If uncertain, do it…just to be safe* (W1F)<br>• *There’s always the fear of losing patients. ..If we don’t treat, I’m uncertain, if I don’t treat my patient will die. So I treat until I get a test result and I’m sure* (W1F)
	Fear of malpractice and litigation	• *Maybe something goes wrong and then you will face litigation, guidelines, your colleagues, patient/doctor relationship, you will face all of it. Nobody prefer to face these. They just going to stand back. You want the test? Go, have a test. Be my guest. Nobody wants to …stick their neck out* (W1F)<br>• *So the sense of moral obligation…There’s one A&E consultant that refers quite a lot and he will say we’re duty bound to investigate this. And I’ve been thinking then, like where does that sense of duty come from? And I think it comes from their fear of malpractice rather than an intrinsic duty to be a good doctor to do this…And it’s like separating out what are your duties as being a good doctor from what are your duties to protect yourself from malpractice* (W1F)
	Sense of medical obligation	• *Testing can be a part of taking a patient seriously* (W2J)<br>• Personality<br>o *I think for a lot of practitioners there’s fear of being seen as not being…fantastic. Not being good…we’re these very much hyper-performing type A kind of people that don’t take mistakes well* (W1F)<br>o *A lot of doctors have become doctors and are successful getting into medical school ‘cause they’re pretty obsessive…as a group maybe our tolerance of (?) testing isn’t fantastic* (W2J)<br>o *Just to be able to keep your ego in check so then you can say, actually I’ve just done your ultrasound scan and I haven’t got an answer for you at the moment* (W1F)<br>o *…the fragility of ego, fear of appearing ignorant.* (W1F)
	Clinician knowledge and understanding	• *If I have a young doctor, they have to do more testing than I have to because they are more insecure…so they have to search in the broader field that I…because I’m much more confident…so I can do much less* (W3K)<br>• *A disconnect with what their knowledge and attitudes are and what their practice is able to be…that’s probably an issue* (W1F)<br>• *And I think these sort of things here assume that our education or training is correct. Because sometimes it doesn’t emphasise to the students that what we know is just a medical model. And it’s not the definitive knowledge. So sometimes some clinicians come out thinking that this is fact. And so they base on that. And try to strive for that goal* (W1F)
	Cognitive biases and experiences	• Entrenched habits<br>o *Each clinician’s habits and…default settings, they…people come to me with heel pain. Automatically heel x-ray. And I’ve never second thought because that’s how we’re taught and that’s mainly from years and years it’s entrenched….They’re habits. They’re habits that get adapted to the culture. They get their habits from the*

		culture. That they've been trained to and they've been working there and they break that habit with them if they move to other organisations (W1F)<br>○ *So there was this thing about routine. Because it's very hard to stop people from doing like (ECG?) with…(?) that's really hard to stop. Really hard. The patients want to do it. The nurses want to do it. It's done. Like you, you arrive to see the patient that the nurse has already worked up and it's been done…You have to look at it* (W2J)<br>• Personal experiences<br>○ *When something touch you because it's something you have experience…something you have a fear about yourself…then you suddenly get…the uncertainty is very difficult. And if something which don't worry you it's much easier to be…to accept uncertainty* (W3K)
**INTERPERSONAL FACTORS**	Pressure from medical culture	• Expectation to label<br>○ *I think there's always a pressure to diagnose with something, come up with a name or a label for your condition. It's not good enough to say we don't know what you're suffering from. It's not good enough. So they have to sort of conjure up some kind of diagnosis* (W1F)<br>○ *For some conditions like sciatica, for any kind of pain…People always try to ram a square peg into a round hole. You know, so it must be sciatica so we can find every way to justify our diagnosis …because that's easy to prove, sciatica, rather than anything else, so we better take this option* (W1F)<br>• Intolerance of making mistakes, over responding to mistakes<br>○ *So I had something in a discrepancy meeting…And the first thing that happened when I got the email was this sense of I'd done something wrong. And then the second thing that happened was, ok, well how am I going to change my practice now?..I missed that thing, so now I need to scrutinise that thing and if I'm not sure I should maybe draw people's attention to that thing that might be there…There was no advice on like how you cope emotionally when you made a mistake and then how you actually keep your even keel of your practice so you don't tip into like over calling stuff from then on in* (W1F)<br>○ *Learning versus performance…doctors often over responding to mistakes* (W1F)<br>○ *I can feel like a good doctor for about five days. Then something goes wrong, you know, you're on this knife edge and you fall off. And then you make a minor or major, whatever…and then you overcompensate, you inevitably overcompensate…and that never gets any better* (W2J)<br>• Blame culture<br>○ *I think within medicine there's a bit of a blame culture…amongst peers…and there's that to, sort of protecting yourself from colleagues in a sense…failure to diagnose…would certainly push towards overdiagnosis* (W2J)<br>• Existing workplace culture<br>○ *Medical doctors they train to choose wisely but they come to this organisation, the culture is to order the test, do this, do that. And they need to be with that culture otherwise they're going to be the bad apple who is going to be pushed away* (W1F)<br>○ *They want to fit in the accepted culture.*<br>○ *You've got all these pressures because the environment that you work in has a culture…that is not easy to define because there are so many different things (?) into it. And that culture is an influential thing* (W1F)<br>○ *If they don't want to go for more tests they are self-confident to say, you don't need more tests. (But) At the end they're going to be the bad apple in that organisation. Who is not doing what the organisation is going to do* (W1F
	Pressure from colleagues	• Personal practice preference overridden by seniority/hierarchy

		○ *My daughter…she said her boss, even though she has, she intuitively doesn't think a CT scan. The boss will say get one done* (W1F)<br>○ *They're (junior doctors) having conversations with me going, I really didn't think we needed to do this but now I've run it past the boss and they want it…it's almost like they wished that they hadn't spoken to a senior about it* (W1F)<br>○ *There is a real pressure to…'cause how are you going to get, like justify going against that consultant* (W1F)<br>○ *And (?) in general practice to resists that, because once…has been raised by the specialist…you might think that it's unnecessary, but it's very hard to argue that for the patient because they wouldn't doubt what the specialist said. So specialist just kind of rules, you know?* (W2J)
	Pressure from patients and doctor-patient relationship	• Patient demand, maintaining relations<br>○ *I want those. If you're not going (?), I'll go to him. That's what they do* (W1F)<br>○ *People are willing to pay, patients are willing to pay for MRIs and various things that there's no Medicare rebate for* (W2J)<br>• Dr Google<br>○ *It's probably due to…what's available on the internet…everyone's become global doctors…and I don't know how as clinicians you say, yes, you've Googled it but what you think is this thing..what we know is this* (W1F)<br>○ *The knowledge…clinician knowledge and understanding is having a clash with the knowledge and understanding of, in this case the practitioners are schoolteachers who've been told, this is real. These symptoms are real* (W1F)<br>○ *Social media is really big in terms of creating new diagnoses and people are applying those to themselves. And then coming to…I've got this, I need to have that…I find those…hard to push back against* (W2J)
**ENVRIONMENT/COMNTEXT**	Pre-emptive testing for subsequent care	• Validates hand over<br>○ *You don't know the actual doctors at the hospital. Eight hours later and it's a new doctor and if they prefer not to only rely on what you have expressed in the medical history but also the other things, so they can say ok, I do rely and I do trust their approach to this patient based on all the different kind…that are used…especially when they know it's a young doctor…even though I might be sure about the medical history, what is actually the right thing to do…makes your hand over, I guess, sort of validated* (W2J)<br>• Fragmented care<br>○ *The other thing that drives a lot of testing…at least increased testing is just the fragmentation of care and the poor communication between providers. Tests just get repeated so often…and patients go along and don't ask…Someone said I had to* (W2J)
	Availability and ease of access to tests	• *We installed a new MRI scanner to cope with the demand for MRI, but what happened? We just got more requests for MRIs* (W1F)<br>• *Different molecular tests, new technologies, today are available every hospital or near to hospital…the number of test (unclear) asking for…specialists use more…more specialists like to catch all the goldfish…you have molecular available? Yes. You try and ask for a lot of molecular tests just to find one disease. And this is increasing, the number of blood tests…The availability is very important…If you have it a lot, you use a lot* (W2J)<br>• *Every GP is, we're co-located, pathology's on site, X-Rays next door, it's all kind of in your face. It's not hard to get these tests* (W2J)
	Time constraints, physical vulnerabilities, language barriers	• *I think the most common reason in my practice for doing a test that's probably marginally unnecessary is to buy myself some time. You know, so that if you can do a cheap test that gives you another week for the patient to get better on their own… you want to use time as a diagnostic tool* (W2J)

		• *I think it's a time pressure thing … You cant get to the specialist to ask the question, so you just go 'll do it, even though I might not agree with it. I might not think it's necessary. Pragmatism dictates that* (W2F)<br>• Physical vulnerabilities - The human factor<br>○ *Really basic stuff, like being tired and not having enough to eat and you don't want to have another battle because you cannot make the decision* (W1F)*…it's like you haven't had your break…it's meant to be one of the first cognitive functions that goes, is your ability to tolerate risk if you're tired and stressed…*<br>○ *Sometimes the throw away line's just to, you know, as I say, overworked, tired and stuff like that. And just have a quick solution and just go. Rather than a real time there to investigate what's the underlying. So time poor and overworked, stuff like that. Attitude…the human factor…all clinicians are human* (W1F)*…I mean in those situations why wouldn't you just go for the test or why wouldn't you just go for the let's get this patient sorted?...that whole umbrella of factors …provide greater understanding of what really is going on* (W1F)
	Guidelines, protocols, policies	• Inconsistent make it difficult to know what is the current advice<br>○ *These state guidelines might be different from the other state guidelines. And they can all be different from GP guidelines. And so the guidelines do not always point out the same thing* (W1F)<br>• Encourage more testing, not less<br>○ *Guidelines very rarely tell you when it's ok not to do something. The guidelines will always be a positive – do this, go out and do this. Guidelines always are being to do this test.* (W1F)
	Financial incentives and ownership of tests	• Renumeration, diminishing returns<br>○ *It takes time to explain things…it's a real problem because…the amount of renumeration…GPs are funny, they have to maintain their income is what I'm trying to say …so volume wins in terms of income. Time loses* (W2J)<br>• Systems operating outside of health care – seeking diagnoses for funding<br>○ *The line-up to visit the paediatrician now is coming from parents being sent to get a diagnosis by school teachers (ADHD, autism)…and the frustration is that the parents will say but I've been told I can't go back until I've got some Ritalin… it's probably really strong in terms of a push from the school, because if they get the diagnosis the schools get funding* (W1F)<br>○ *If you've got a child who's…that may be a bit disruptive and disorganised and needs some assistance in the classroom, they'll get it if they've got the diagnosis…Please label my child as autistic so they can get the help they need…If they can get that diagnosis quickly they'll get the help quickly* (W2J)<br>○ *The doctors are the people that sign the forms for all sorts of stuff…But the teachers often initiate it these days* (W2J)<br>• Care plans, quality measures<br>○ *I find three months for a lot of people is way too early. They don't need it. But there's an expectation that they get a review of a problem and they get a repeat of all the tests that they had at the initial…an unintended consequence of that funding, that the government policy I think has probably led to an increase in use of pathology testing, particularly.* (W2J)<br>○ *Measuring quality mandated, you know, that you want to get blood pressure and blood sugar down to a certain level, therefore you have to do these tests all the time till you got a good one...all sorts of gaming goes along and that definitely drives more tests…and we're moving towards outcomes based payments. So we have to repeat what you've done'* (W2J)<br>○ *There's another standard test..the over 75s…health assessment. .. And I have to remind the practice nurse, please tell my patients don't do a urine analysis unless there's a problem…and then they also get an ECG done* (W2J)

	Contemporary medical practice and new technology	• Decline in patient history taking<br>○ *Medical doctors are losing your clinical aspects. (rolling?) to the patients. And replace clinical (?) by test…Yeah, I sometimes think that real scattergun approach to investigation is because doctors are very busy and they don’t take the history…they just do the test.* (W2J)<br>○ *I think young doctors often have…do not have the confidence in their examinations and history taking skills that they should have…so they rely on tests…you don’t seem to be creating new doctors with that confidence to rely on…*(W2J)<br>• *Different new diagnoses and how they are defined by data and measurements instead of actual physical manifestations* (W2J)<br>• *A typical expression of diseases and so on has been changed to objectify. This comes where you have to be able to quantify stuff. You have to be able to get it confirmed by other people as well.*(W2J)<br>• Attitudes and conditioning of modern society<br>○ *I want this and I want this now. But it’s not like intolerance of uncertainty but it’s an intolerance of waiting to see how things declare…that might be more of a cultural fast paced beyond medical practice* (W1F)<br>○ *Our human culture has changed…so our treatment culture has to change accordingly as well to adjust to it.* (W1F)<br>• *If I want to know something I just get on the phone or I Google. And I can do that now. I don’t have to wait til later…and I think that’s…patients they want to know…give me the test, give me the answer…it’s really unfair. Because I think, how do you learn to (?) that it’s possible to survive uncertainty, if it’s…pretend or think you’ve found certainty within 30 seconds …so I think it’s a generational thing…And it may seem very convenient, but actually…it’s also a burden* (W2J)
